# Labels on Levels: Labeling of Multi-Scale Multi-Instance and Crowded 3D Biological Environments

**DOI:** 10.1109/TVCG.2018.2864491

**Published:** 2018-12-09

**Authors:** David Kouřil, Ladislav Čmolík, Barbara Kozlíková, Hsiang-Yun Wu, Graham Johnson, David S. Goodsell, Arthur Olson, M. Eduard Gröller, Ivan Viola

**Affiliations:** TU Wien; Faculty of Electrical Engineering, Czech Technical University in Prague; Masaryk University; TU Wien; Allen Institute for Cell Science; The Scripps Research Institute; The Scripps Research Institute; TU Wien and the VRVis Research Center; TU Wien

**Keywords:** labeling, multi-scale data, multi-instance data

## Abstract

Labeling is intrinsically important for exploring and understanding complex environments and models in a variety of domains. We present a method for interactive labeling of crowded 3D scenes containing very many instances of objects spanning multiple scales in size. In contrast to previous labeling methods, we target cases where many instances of dozens of types are present and where the hierarchical structure of the objects in the scene presents an opportunity to choose the most suitable level for each placed label. Our solution builds on and goes beyond labeling techniques in medical 3D visualization, cartography, and biological illustrations from books and prints. In contrast to these techniques, the main characteristics of our new technique are: 1) a novel way of labeling objects as part of a bigger structure when appropriate, 2) visual clutter reduction by labeling only representative instances for each type of an object, and a strategy of selecting those. The appropriate level of label is chosen by analyzing the scene’s depth buffer and the scene objects’ hierarchy tree. We address the topic of communicating the parent-children relationship between labels by employing visual hierarchy concepts adapted from graphic design. Selecting representative instances considers several criteria tailored to the character of the data and is combined with a greedy optimization approach. We demonstrate the usage of our method with models from mesoscale biology where these two characteristics—multi-scale and multi-instance—are abundant, along with the fact that these scenes are extraordinarily dense.

## 1 Introduction

New technologies and advancements in computing enable us to capture and model enormous amounts of data, describing complex multi-scale structures and processes, and store them as 3D scenes. One example of complex multi-scale structures are molecular biology models. These models represent complex assemblies of proteins where the proteins themselves are composed of polypeptide chains that in turn are composed of atoms. In this case, the scale levels are the atomic, polypeptide chain, protein, and protein assemblies levels.

Complex multi-scale scenes often contain millions or even billions of 3D objects. Current capabilities of modern GPUs and level-of-detail techniques enable users to explore such scenes interactively, and smoothly transition between several scale levels [29]. Each of these levels shows the objects in the scene with a particular complexity, based on the distance of the object from the observer.

The multi-scale aspect of the scenes makes visual communication of their structure a challenging task. Recently, several approaches that improve the visual communication of structure of multi-scale scenes appeared. Hun et al. [21] visually communicate the parent-child relationship in a multi-scale hierarchy by deforming the multi-scale scene. Waldin et al. [50] utilize a dynamic approach to color objects in multi-scale scenes based on several levels of the hierarchy to better distinguish between structures on various scale levels.

On the one hand, through visualization we are able to visually communicate spatial arrangements of the objects and, to a certain extent, their multi-scale hierarchical relationships. On the other hand, visualization cannot convey the details that are typically covered by a text description. Therefore, to ease the understanding of multi-scale structures, we need to interconnect the visualization of the complex structure with a textual description.

In this paper, we present an approach that uses labels, i.e., textual annotations of the objects, to mutually interconnect the visualization and the textual description. The labels are positioned over the visualization of the multi-scale scene in a manner that associates them with the depicted objects. For these scenes, the labels should reflect their multi-scale aspect. In other words, we should label the objects in a scene taking scale and hierarchical grouping levels into account.

Multi-scale labeling introduces several new problems that have not been addressed by existing labeling methods. For each view of the scene, we need to select appropriate scale levels on which we will label the objects. One view can contain objects from different scale levels and the labeling technique should take this into account. Changes in the scale level of the objects should induce corresponding changes of their labels as well.

**Fig. 2:**
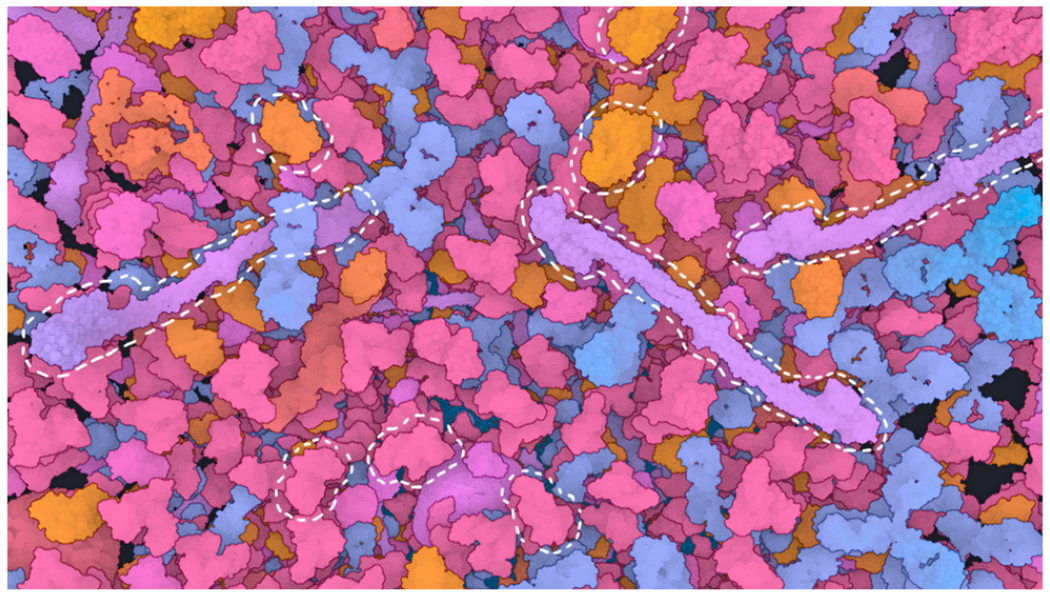
Blood plasma as an example of a biological environment. Very many instances of just a few protein types make up a dense scene. To illustrate this, three protein types and three instances each (of many) are highlighted.

Complex multi-scale scenes usually contain many objects and not all of them are unique. In our exemplary biological environment, a blood plasma can be a typical representative of such a scenario. Blood plasma is a component of blood, consisting of proteins and other molecules and ions in very dense concentrations. However, the number of distinct molecule types is very limited, and blood plasma contains many instances of the same molecules (as can be seen in Figure 2). Typically, there is no need to label all instances of each type, but only one or several representative instances, e.g., representative proteins of each protein type. Such multi-instance labeling requires an automated selection of the representative instances, which is not addressed by existing labeling methods.

Complex multi-scale and multi-instance scenes are typically very crowded, i.e., densely populated with objects. This holds especially for molecular biology models. Therefore, there is very little or no space where the labels can be placed without overlapping the objects in the scene. This is the case especially if the camera is close to the objects in the scene.

In this paper, we introduce a novel solution to the problem of **multi-scale** and **multi-instance** labeling, which is applicable to arbitrary domains. We present a conceptual framework for interactive labeling in densely populated multi-scale and multi-instance environments, where annotation on several scale levels is possible and desired. The framework allows users to interactively explore complex multi-scale and multi-instance environments. It labels representative instances of objects in the environment with respect to the current viewpoint and the multi-scale hierarchy of the scene. Most importantly, the presented method works in real-time, which allows users to interactively explore the environment. The main contributions presented in this paper are:

- A conceptual framework of labeling in environments with novel characteristics not yet considered in previous work.
- A real-time algorithm for choosing the appropriate label level for each object in the scene. The approach allows us to label the same object type on several different scale levels.
- A real-time algorithm for selecting representative instances of a given object type for labeling. The representative instances are chosen according to visibility and position with respect to the other objects of the same type in the scene.
- A description of a label transition that happens when the scale level changes. The label transitions help the users to maintain their orientation in the multi-scale hierarchy.
- An approach to make the movement of labels temporally coherent during user interaction. This is accomplished by re-projecting world space positions of the labels and at the same time biasing the algorithm towards previous label position.

We demonstrate our solution on a scene of a human immunodeficiency virus (HIV) in blood serum [24,25] from the domain of molecular biology. The scene is multi-scale, where the highest level contains the entire HIV virion, consisting of a lipid bilayer on its boundary and an inner compartment, containing capsid and many proteins. The capsid envelope consists of pentameric and hexameric molecular complexes and the inner part of the capsid contains proteins and a RNA fiber. Each of these structures are composed of atoms. The building parts (i.e., lipids, proteins, complexes) occur in the scene many times, making this an ideal exemplary scene for our multi-instance labeling approach. The scene is very densely populated with the objects. Despite showcasing our approach on a scene from the molecular biology domain, we believe that our approach can be utilized in other domains with similar properties as well.

## 2 Related Work

Methods for positioning labels, i.e., short textual annotations, in two- and three-dimensional scenes have been intensely investigated for decades. The general goal of labeling methods is to algorithmically create aesthetic and compact label layouts where all labels are readable and unambiguously associated with the labeled objects.

### Labeling of 3D scenes

Labeling of 3D scenes, i.e., positioning of textual annotations in 3D space, is utilized mainly in the medical domain [33] where both the spatial relations between the objects and their semantic description is needed to understand complex structures and processes in a human body.

In general, labeling approaches typically operate in the screen space on the projected image of the 3D scene. We distinguish between two types of labels. *External labels* are placed in the free space around the projected 3D objects and are connected with the projected 3D objects through leader lines. *Internal labels* are placed directly over the projected 3D objects or are just touching them.

We organize the external labeling approaches according to their strategies to localize the free space around the projected 3D objects. Preim et al. [38] and Huang et al. [22] enclose the projection of the 3D scene with an axis-aligned rectangle and position the labels in the free space outside this rectangle. Ali et al. [1] and Hartmann et al. [18] enclose the projection of the 3D scene with its convex hull and position the labels in the free space outside of the hull. Later, Čmolík and Bittner [11] provided a GPU implementation of this approach. Positioning the labels around the convex hull instead of the bounding rectangle results in a more compact label layout. An even better result can be produced if the labels are positioned in all the free space around the projected 3D objects. Stein and Décoret [46] presented a greedy algorithm that positions the labels in the free space around the projected 3D objects. The algorithm evaluates whether the labels fit into the free space using a summed area table [19] and uses shadow regions to prevent the overlapping of labels.

The main limitation of all mentioned external labeling approaches is that they require free space around the labeled objects in the 3D scene or around proxy objects bounding the projection of the 3D scene. This precondition cannot be expected in very dense scenes, such as in our molecular biology environment. For such dense 3D scenes, internal labels placed over the projections of the 3D objects are more suitable. Bell et al. [5] introduced the very first algorithm for internal labeling. They approximate each projected 3D object by the smallest axis-aligned enclosing rectangle. The approach determines the free space based on the rectangles. It places each label at the center of the largest rectangle contained in the projected 3D objects that is not occluded by any other rectangle. The size of the labels is determined by the free space available and predefined constraints. If a label cannot be placed internally, it is placed externally in the free space outside of the object and connected with the object by a leader line. Other approaches [14–16] directly use the projected 3D objects and their visibility to evaluate the free space for internal and external labels. The internal labels are positioned based on the skeletons of the projected 3D objects.

Internal labels are also adjusted according to the shape of the labeled objects. Mass and Döllner [30] use object-integrated billboards to position labels over rectangular 3D objects, such as buildings. Ropinski et al. [40] approximate the 3D objects with Bezier patches and align the labels with the geometry of the underlying structures. Cipriano and Gleicher [10] provide a solution for surface regions with high curvature, where the surface-aligned labels would be distorted. They create scaffold surfaces onto which the labels are placed.

Prado and Raposo [37] internally label 3D objects with spherical, cylindrical, and rectangular shapes in object space. The occlusion of the labels by the 3D objects is not considered, but the labels are positioned several times on each 3D object.

None of these techniques deal with a multi-scale situation, i.e., the presence of objects on different levels of detail and corresponding levels of labels. To the best of our knowledge, the only 3D scene labeling approach that considers multiple scales was presented by Götzelmann et al. [17]. The approach takes into account only two scales. The labels are organized into several groups and in each group they are positioned close together outside of the convex hull of the projected 3D scene. Possible crossings of the leader lines between different groups are not resolved.

### Scale-Aware Labeling in Cartography

Although placing name labels is essential in cartography and has been extensively investigated in geovisualization [9, 23, 45, 51], labeling multiple objects in multi-scale environments is studied separately. Online map services usually place anchors to emphasize objects of the same type along different zoom scales, since the positions of objects are predefined and can be easily computed. Lin et al. [27] introduced *Many-to-One Boundary Labeling*, where they placed exactly one external label for objects of the same type along the boundary of the map domain. This has been extended to study placements without crossings [28] and along specified backbones [4], but no scale-dependent approach has been proposed.

Initially, researchers studied static map-labeling approaches, which are insufficient for navigation purposes due to their high computational requirements [7]. To solve this, fast dynamic labeling techniques were developed for moving and zooming in/out of the map domain. Acceleration is achieved by decomposing the map content into several scales and only the significant objects will be annotated [8]. Nonetheless, scales were mostly handled independently and thus visual consistency was often ignored [12, 32]. Poon and Shin [36] developed the pioneering approach to build a hierarchy tree to guide label placement for user navigation. Been et al. [2, 3] then extended the shape and orientation of labels to develop active range optimization (ARO) for general dynamic labeling purposes. The idea is to maximize the persistence of name labels by computing placement conflicts using rectangular pyramids in the zoom space. Zhang et al. [54] extended pyramids to trapezoidal boxes to constrain lower and upper bounds of labels along zoom scales. Wu et al. [52, 53] introduced the hierarchical structures of datasets into the label placement process. This is done together with optimizing the label movement and the leader lines to improve labels at each scale. Other researches focused on slider-based [44] and 2D rotation-aware [13] label placement, while artifacts usually occurred during exploration. Slider-based labeling uses labels that slide along their anchor points. Rotation-aware labeling approaches determine an optimal label placement that is consistent upon a 2D rotation of the map.

Unfortunately, these scale-aware techniques cannot be easily adapted to the labeling of 3D scenes, because they do not consider camera rotation with three rotational degrees of freedom. Further, there is no unambiguous definition of the level-of-zoom in 3D environment for a whole rendered frame. In contrast to scale-aware labeling of interactive maps where the level-of-zoom is defined from the sea level, we cannot define the level-of-zoom as a variable with one degree of freedom for 3D scenes as the distances of 3D objects from the camera are not constant.

### Labeling in the Visualization of Biological Environments

Labels are widely used in molecular and mesoscale visualizations, but with significant limitations. Many effective molecular graphics programs are available, such as Jmol/JSmol [20], Chimera [34], and PyMol [43]. They are essential tools in structural biology research and outreach. Two types of labeling are implemented in these tools.

First, information about individual atoms, including atom names and corresponding amino acid and polypeptide chain, may be obtained by hovering over an atom in the rendered frame. In our experience, this is indispensable in day-to-day research if combined with flexible scripting and coloring tools.

**Fig. 3:**
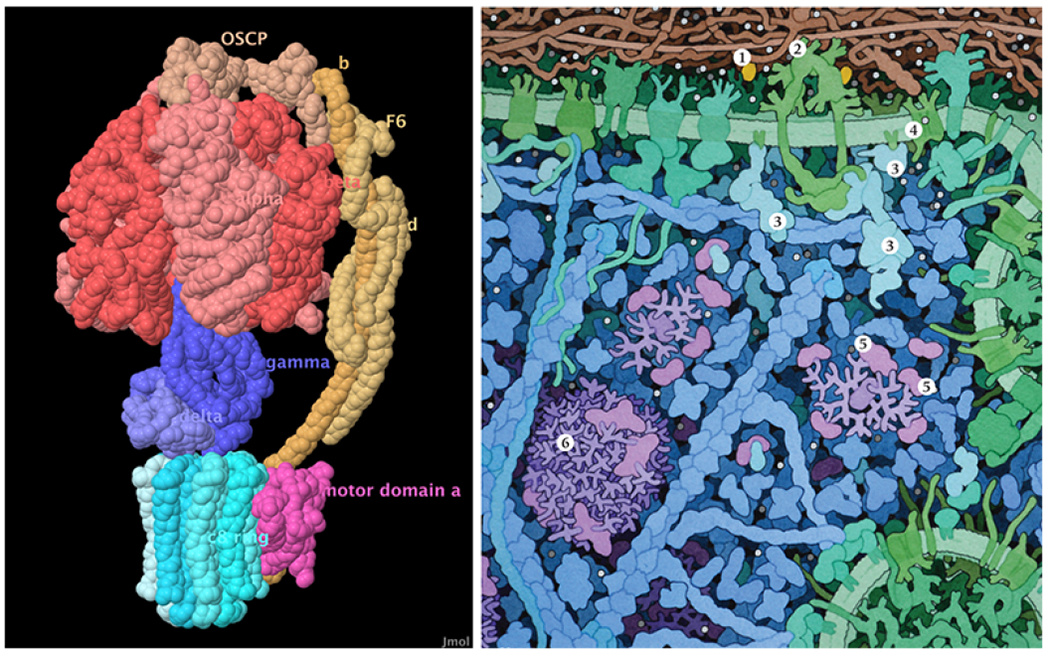
Left: JSmol image of ATP synthase, with subunits labeled. Atoms were chosen manually for the placement of each label. With the default settings, labels are occluded by the spherical atoms, so several labels are not visible in this view. Labels are colored based on the colors of the subunits. Right: Detail of a poster presented at the educational portal of the RCSB Protein Data Bank, showing molecular processes in insulin signaling. An accompanying caption is used to identify each molecule (1-insulin, 2-insulin receptor, 3-signaling proteins, 4-glucose transporter, 5-glycogen biosynthetic enzymes, 6-glycogen).

Second, 3D labels may be attached to user-defined atom positions. A variety of customizable parameters, e.g., color, font size, offsets, shaded backgrounds, and different approaches to occlusion, allow users to optimize the frame. Problems with overlap and visibility continue to plague these types of labels in all but the most simple applications. Figure 3 (left) shows an example of labeling in JSmol. In this case, considerable time is required to manually pick appropriate atoms for placing labels and to reduce occlusion problems while interactively manipulating the object.

There is also a long history of labeling 2D atomic, molecular, and cellular structures, such as those used in textbooks and professional publications. These examples may be used as style guides when designing interactive labeling approaches. The labels are typically added to the imagery in a post process, allowing the designer to optimize the placement and other characteristics. Effective designs often include approaches like drop shadows or outlines to provide contrast between labels and the image. Furthermore they use blank space (if present) around the main object, and add line breaks or abbreviations to the text to reduce the horizontal extent of labels. Static images also provide the freedom to place most of the label information into an accompanying caption or legend, using simple reference numbers or letters to identify features in the image itself (see Figure 3 right). With static printed images, users are typically willing to devote more time for switching attention between figure and caption, in a way that would be prohibitive for interactive applications with continually changing views.

In addition, complex static images are often labeled with numbers or letters to keep occlusion low (see Figure 3 right). The label serves as reference to text near the illustration called a “key”, which contains the label and often a more extensive description of the object. This is one of the typical labeling scenarios used especially in 2D static images and scenes. However, this solution is not feasible for dynamic and complex 3D scenes as the user constantly needs to switch attention between observing the label and its description.

In case of interactive environments, we can afford to place the entire labels directly into the scene, close to the position of the corresponding object. This also fits the recommendation of Tufte [48] to place labels directly on the graphic itself, without legends, and close to the labeled features. This avoids using the leader lines between the features and their labels. The user can temporarily turn off the labels and examine the structure itself, which solves possible occlusion problems.

## 3 Labels on Levels – Method

**Fig. 4:**
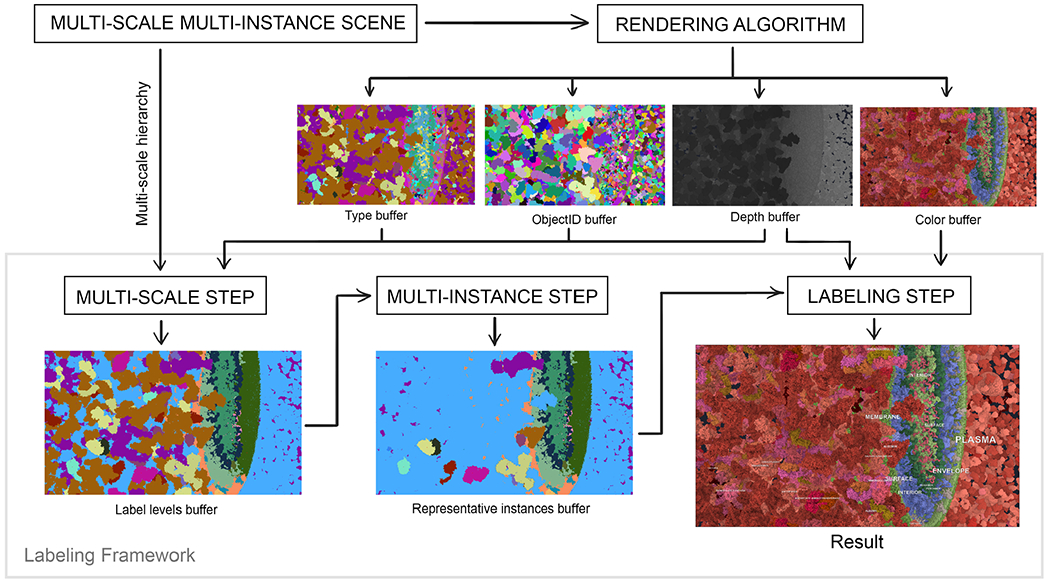
Overview of the Labels on Levels (LoL) framework. The multi-scale step utilizes the G-buffer result of a rendering algorithm with the scene’s hierarchy in order to generate depth-aware regions that are to be labeled. Afterwards the multi-instance step selects a representative instance together with a label anchor point inside the chosen instance, which is optimal according to specified criteria.

In this section, we describe our method of labeling multi-scale and multi-instance scenes. The method is designed to work as a post-processing step, performed after the scene has been rendered, to make it easily adaptable for use with various rendering frameworks and application scenarios. The overview of our approach is depicted in Figure 4.

To render the scene, we use the approach of Le Muzic et al. [26]. Their technique allows us to render the complex multi-scale and multi-instance scene in real-time by employing a level-of-detail scheme that simplifies the shapes of proteins if they are far from the camera. We have modified the approach to produce a G-buffer [41], consisting of a Type buffer, an ObjectID buffer, a Depth buffer, and a Color buffer. All these buffers, together with the multi-scale hierarchy of the scene, are the input to the Labels on Levels (LoL) method (see Figure 4).

The multi-scale hierarchy is supplied as a scene graph, commonly used in various 3D applications and game engines. This tree-like structure contains nodes that represent parent-children relationships between scene objects. The leaves of the hierarchy represent objects of the lowest semantic level in the scene. In our exemplary scene containing the HIV virion, the leaves correspond to proteins. The inner nodes of the hierarchy contain objects on higher scale levels composed of objects from lower semantic levels down the tree structure. Each object in the multi-scale hierarchy has a unique objectID and a type assigned. For example, if there are several instances of a certain protein type in the hierarchy, then each of these instances will have a unique object ID, but they will be of the same type. In our method we use the type information at the nodes in order to label only one representative instance of a particular object type.

The multi-scale hierarchy also expresses the spatial characteristics and extent of this particular type of data. The higher a node in the multi-scale hierarchy of the scene is, the larger the structure annotated by the corresponding label becomes. A parent node occupies more space in the scene than each of its child nodes, because the parent node is composed of its child nodes and this should be reflecting in the labeling as well.

The four buffers of the LoL approach contain various information necessary for the labeling. The Type buffer contains the encoded type of each protein projected onto the screen. The ObjectID buffer contains unique identifiers of the leaves of the hierarchy (in our case individual proteins) projected onto the screen. The Depth buffer contains the distance to the camera for each protein projected on the screen. The Color buffer contains the color of each projected protein. All buffers are implemented as 2D textures.

Our goal is to find a set of textual labels that annotate the object instances which are the most visible in the scene. At the same time, the labels should not visually clutter other objects in the scene. In addition, the movement of the labels during user interaction with the scene should be temporally coherent, i.e., the label positions should not change abruptly between frames.

We address the visual clutter by utilizing two characteristics of the data. Instead of labeling all visible protein instances in the scene, we label only selected instances on the appropriate scale levels. Our labeling framework consists of three steps:

- In the *multi-scale step*, we group pixels based on the type of the visible instance that occupies it, the distance of the instance to the camera, and the multi-scale hierarchy. After this step, each region in the output represents an instance of a specific object type on a common scale level.
- In the *multi-instance step*, we choose one instance of each object type that corresponds to the best representative for each scale level. In this step, the anchor point for the label is also chosen.
- In the *labeling step*, we label each representative instance and draw the label into the color buffer, which contains the already rendered scene. We are using internal labels that are placed in the 3D object space. Label positioning, as described in the related work, is only a small part of our method.

In the following sections, we discuss these three steps in detail, describe our approach to make label movements temporally coherent, and present our approach to the label rendering.

### 3.1 The Multi-Scale Step

The general idea behind the multi-scale step is to group objects further from the camera and label them as a group using a representative group name, rather than labeling separate objects. The distance to the camera is used to determine the appropriate level for labeling of an object in the multi-scale hierarchy. In consequence, individual objects, such as proteins, that are far away from the camera will not be labeled individually. This approach allows us to reduce the number of labels, and, at the same time, to communicate to the user in a single image the multi-scale hierarchical relationships in the scene.

**Fig. 5:**
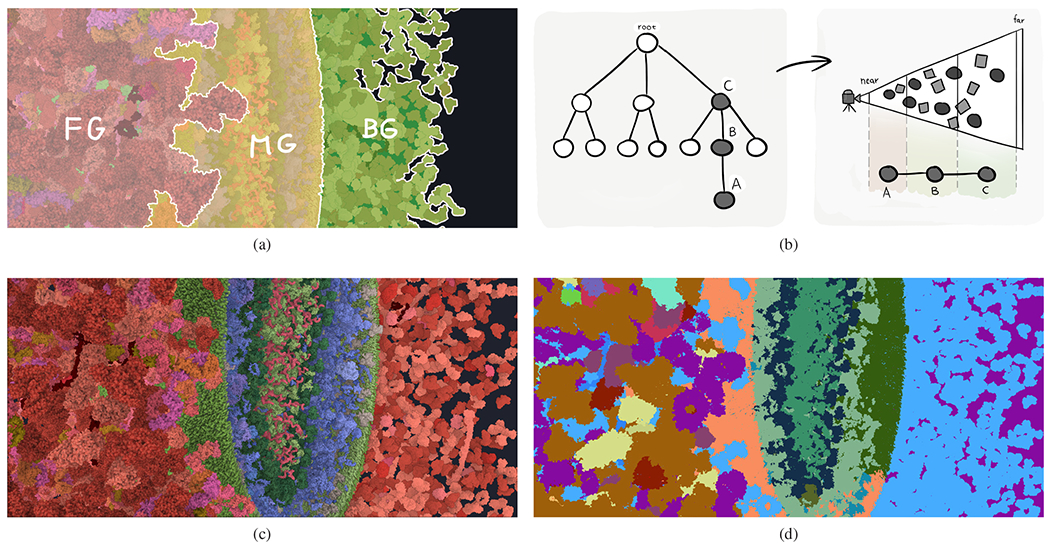
(a) Illustration of subdividing the scene into several scale levels (foreground, middle-ground, background). (b) The distance of objects to the camera determines which nodes of the multi-scale hierarchy are labeled. (c) A typical camera view in the HIV scene. (d) Result of the multi-scale step for the view in (c). Here the colors indicate different object types in the Label levels buffer.

Figure 5a illustrates the approach. In this sketch, the scene is sub-divided into three regions based on the distance of the objects to the camera. In the foreground (FG, red), individual molecules will not be grouped into higher-level structures and the representative instance of each visible molecule type will be labeled. In the middle-ground (MG, yellow), the HIV virion proteins will be grouped into higher-level structures (e.g., membrane, inner matrix, and capsid) and the representative instances of these higher-level structures will be labeled. In the background (BG, green), all blood plasma proteins will be grouped into one top-level structure and annotated by a single label as *blood plasma*. In order to achieve this labeling, we propose the following algorithm.

The input to the multi-scale step is a G-buffer consisting of a Depth buffer, an ObjectID buffer, and a Type buffer. These buffers are determined during the rendering of the scene An additional input to the multi-scale step is the scene graph, which contains the multi-scale hierarchy of the scenery. The input G-buffer contains depths, object IDs, and types of the objects at the lowest scale-level, which are stored at the leaves of the multi-scale hierarchy.

The output of this step is the Label levels buffer, a G-buffer containing the distance of objects to the camera, instance IDs and types of the objects represented by nodes of the multi-scale hierarchy at the determined labeling levels.

In the preprocessing step, we calculate the number of nodes on the path from each leaf node to the root in the multi-scale hierarchy. The appropriate labeling level is determined for each pixel of the input G-buffer in parallel. The algorithm for one pixel works as follows:

#### Step 1

We obtain the distance to the camera, the unique object ID, and the type from the input G-buffer.

#### Step 2

Based on the object ID, we divide the [0, 1] depth range into *n* uniform intervals, where *n* is the number of nodes on the path from the object node to the root of the multi-scale hierarchy (see Figure 5b).

#### Step 3

We traverse these intervals *I*_0_, *I*_1_, *I*_2_, …, *I*_*n*−1_. For each interval *I_i_*:

1. We test if the distance of the visible object at the pixel to the camera (in the [0,1] range) is in the interval *I_i_*.
2. If the distance is not in the interval *I_i_*, we continue with the next interval *I*_*i*+1_. We take the interval *I_i_* if it is the last one.
3. Otherwise, we return the *i^th^* node (its instance ID and type) on the path from the leaf to the root as the appropriate labeling level for this pixel.

The output Label levels buffer has the same size as the input and for each pixel contains the instance ID and the type of the object (node) determined by the above algorithm. Further, we store the distance of the object to the camera in the Label levels buffer to speed up reading of the data in the multi-instance step. An example of the result of the algorithm for the scene in Figure 5c is depicted in Figure 5d. Here the objects in the scene are colored according to their types.

### 3.2 The Multi-Instance Step

In the multi-instance step, we are searching for the representative instance for each object type in the Label levels buffer. We are evaluating the pixels of all instances of each object type according to various relevance criteria described below. For labeling we are choosing the instance covering those pixels that satisfy these criteria the best. We use the position of the most suitable pixel for the anchor point. The anchor points will then be utilized in the labeling step to place the labels. The input for this step is the Label levels buffer, containing the instance IDs and types of objects on the determined labeling levels together with their distances to the camera. The output of this step is the Representative instances buffer, i.e., a 1D texture where each texel contains the information about a label placement in the image for one type of object each. In the color components of the texel, we store the instance ID and type of the determined representative instance that will be annotated. The buffer also contains the 2D screen-space coordinates of the labels.

We evaluate the pixels according to four criteria:

#### Saliency criterion *C*_1_:

We use internal labels to annotate the objects in the scene. To allow the users to easily associate the label with the labeled instance, we determine the instance that is the most salient, i.e., the visibly most prominent, one. In such a case, the internal label is more likely to be completely inside the projection of the labeled instance and will not occlude other objects in the scene. We model the saliency of a pixel with respect to an instance as the image-space distance from the instance silhouette. This corresponds to the largest non-occluded segment of the projected instance onto the image plane

(1) $$C_{1}=dist\left(\vec{p},S\right),$$

where $\vec{p}$ is the position of the pixel, *S* is the silhouette of the instance, and the function *dist* returns the shortest distance of the pixel from the silhouette in image space. A pixel farther away from the silhouette is more salient than one closer to the silhouette. The instance with the most salient pixel is then the most salient instance.

#### Distance criterion *C*_2_

Generally in labeling, one of the main requirements is that the labels should be clearly readable. As we are positioning the labels directly in the 3D space of the scene as screen-aligned billboards, the labels should not be occluded by the labeled instance itself. To achieve this, we need to assign the label to the pixel of the instance that is the closest to the camera. We model this criterion as

(2) $$C_{2}=d_{p},$$

where *d_p_* is the pixel’s distance from the camera in the range [0,1]. This criterion penalizes the pixels of an instance that are further away from the camera. Again, the instance containing the pixel closest to the camera itself is also the closest one to the camera.

#### Border criterion *C*_3_:

If a label is close to the border of the screen then part of it may be outside, making the label unreadable. To avoid this, we want to assign the label to an instance that is not close to the border of the screen. First, we calculate the minimal distance of the pixel from the screen border as

(3) $$d_{B}=min\left(x,1-x,y,1-y\right),$$

where we assume that the screen is transformed to the unit square and (*x, y*) is the position of the pixel on the unit square screen. We model the border criterion as

(4) $$C_{3}=\{\begin{array}{c} 1:d_{B}\geq T_{B}\\ d_{B}:d_{B}<T_{B}, \end{array}$$

where *d_B_* is the distance of the pixel from the border of the screen and *T_B_* is a distance threshold. All pixels closer to the border of the screen than the threshold *T_B_* will be penalized. In our implementation, we use the experimentally determined value *T_B_* = 0.2. However, this value can be adjusted by the user. The optimal value of the threshold depends on the size of each label. An automatic calculation of the optimal value for each label will be part of future work.

#### Temporal coherence criterion *C*_4_:

Abrupt changes of label positions during interaction distract the user and require him or her to mentally associate labels with the labeled objects again. Therefore, we reduce abrupt changes by positioning the labels for the current frame close to their positions in the previous frame.

Let us assume that $\vec{p}$ is the position of the pixel, $\vec{a}$ is the anchor point of the representative instance of the same object type as the object type of the pixel in the last frame, and function $\mathrm{dist}(\vec{p},\vec{a})$ returns the image space distance between $\vec{p}$ and $\vec{a}$. The temporal coherence criterion is then modeled as

(5) $$C_{4}=\{\begin{array}{c} \text{1:}\vec{a}\;\text{does not exist in the previous frame}\\ 1-min\left(1,dist\left(\vec{p},\vec{a}\right)\right):\vec{a}\;\text{exists in the previous frame}. \end{array}$$

In consequence, pixels far away from the anchor point of the same object type in the last frame will be penalized, if such an anchor point exists.

Our algorithm for the pixel evaluation works as follows:

##### Step 1

We detect silhouettes as discontinuities of the IDs in the Label levels buffer and store these in a 2D texture.

##### Step 2

We calculate the distances from the silhouettes of Step 1 with the jump-flooding algorithm [39] and store them in a 2D texture. The texture contains the value of the *C*_1_ criterion for each pixel.

##### Step 3

For each object type, each pixel is evaluated according to the criteria *C*_1_, *C*_2_, *C*_3_, and *C*_4_. Criterion *C*_1_ is obtained from the texture created in Step 2. Criterion *C*_2_ is obtained from the Label levels buffer. Criterion *C*_3_ is calculated from the pixel position using Eq. (4). Criterion *C*_4_ is calculated using Eq. (5) from the pixel position and the preceding anchor point $\vec{a}$ obtained from the Representative instances buffer of the previous frame. For each pixel we aggregate the individual, often contradicting, criteria using multi-criteria fuzzy decision making by Bellman and Zadeh [6]. All criteria are modeled as membership functions where the values are always in the range [0,1]. A value indicates the membership of the pixel in a fuzzy set, 0 means that the pixel is not in the set and 1 means that the pixel is entirely in the set. To aggregate the criteria *C_i_* to a single scalar *C* (a higher value is better), we use a non-compensating aggregation with natural fuzzy conjunction. This corresponds to a simple multiplication, where the dissatisfaction of one criterion cannot be compensated by the satisfaction of other criteria:

(6) $$C=\prod_{i=1}^{4}C_{i}.$$

The aggregation of the criteria for pixels where all the criteria are only partially satisfied will yield a higher *C* value than the aggregation of the criteria for pixels where even only one criterion is dissatisfied. This corresponds with our intentions, as placing labels on positions where at least one of the criteria is dissatisfied leads to bad label layouts.

Then, we select the pixel and the instance containing the pixel with the highest *C* value where all individual criteria are well satisfied. This step is done in parallel for all pixels with scattering [42]. The result is stored in the Representative instances buffer, a 1D texture where each texel contains the position of an anchor point, i.e., the position of the pixel with the highest *C* value, for one object type.

### 3.3 The Labeling Step

We utilize internal labels that are placed over the annotated objects. The internal labels are associated with the annotated objects through proximity. Such an association is typically weaker than in the case of external labels, which are explicitly connected with the annotated objects through leader lines.

Our decision has been influenced by two factors. First, densely populated multi-scale and multi-instance scenes do not contain enough free space where the labels could be positioned without occluding any other objects. Second, we see the weaker association of internal labels with the annotated objects as an advantage for structures on higher scale levels, which are represented by the inner nodes of the multi-scale hierarchy. The leader line of an external label points to exactly one position in the image. However, if labeling a compartment composed of dozens of instances of different object types, the leader line will point only at one of the instances. The viewer is likely to associate the label with this particular instance instead of the whole compartment. In such a case, the association can be misleading.

Label placement is done similarly to Bell et al. [5] which position labels over the tagged objects. They position a label at the center of a bounding box enclosing the tagged object. Instead, we position the center of each label at the corresponding anchor point. We obtain the anchor points from the Representative instances buffer produced by the multi-instance step.

The output of the multi-instance step is a 2D position for a label. We now reconstruct the 3D position of the label using the camera parameters and the depth buffer [35].

Each anchor point is a position in 3D and we employ *billboards*, i.e., rectangles in 3D that are always aligned to the camera, as canvases for the labels. The use of billboards positioned in 3D improves the association of the labels with the annotated objects and allows us to make the movement of the labels temporally coherent during interaction. Additionally, the 3D billboards have been received positively by our collaborating domain experts.

### 3.4 Label Rendering

In this section we present our approach to rendering of the labels. We further discuss how the various scale levels are visually communicated through the labels.

We render the labels as camera-aligned billboards. The text is placed on the billboards with texture mapping. In our implementation we are using the FreeType library [49] to access font character data as textures. The center of each billboard is aligned with the position of the associated anchor point that has been obtained from the multi-instance step.

**Fig. 6:**
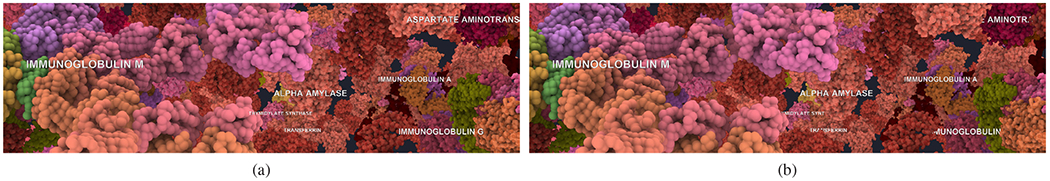
Comparison of the two label rendering modes. (a) The labels are rendered over all objects in the scene. (b) The depth test with the objects in the scene is performed for the rendered labels. The depth test can enhance the perception of the labels as 3D objects and also help with the association between the labels and labeled objects.

We support two modes of label rendering. In the first mode, the labels are placed over the input Color buffer where they are occluding each object beneath. In the second mode, we perform a depth test for a label with all objects in the scene except the tagged one. The depth test is executed based on the information in the Depth buffer. In the second mode, a label can be occluded by the objects in the scene. However, this occlusion enhances the perception of the label as a 3D object, which in consequence helps the user to associate the label with the annotated object. Examples of both label rendering modes are presented in Figure 6.

Labels positioned in one frame are typically tagging objects at various scale levels of the multi-scale hierarchy. Therefore, we need to visually communicate the different scale levels of labels. We apply the concept of a visual hierarchy from graphic design. The size of the labels encodes the scale levels as this naturally implies ordering of the scale levels, i.e., a bigger label is annotating an object on a higher scale level than a smaller label. However, in our case we encounter one complication. As the labels are 3D objects positioned in 3D, the size of the labels is affected by perspective foreshortening. This results in a conflict in the interpretation of the perceived size. The size of the label indicates both the distance from the viewer and the scale level of the tagged object. After discussing with several domain experts, we address this issue by setting appropriate size ranges for each scale level. These can also be adjusted by the user.

Using size to communicate the hierarchy is the most natural option in 2D environments (e.g., text processing), but this is not directly transferable to 3D. Other options, such as different font faces, colors, or glyphs assigned to labels on different levels, are also problematic as they require an explicit mapping to scale levels. It might be beneficial to combine several approaches, e.g., assigning glyphs to different levels in addition to the font size scaling. Further, the hierarchical relationship could be communicated with lines connecting a parent with its children. To avoid visual clutter and edge crossing such lines would only be shown on demand, e.g., during interaction with the associated label.

### 3.5 Temporally Coherent Movement of Labels

In interactive applications exploration is an important component. For labels a temporally coherent movement is crucial. Label placement frame by frame without taking temporal coherence into account can induce abrupt changes in label positions. Even a small change of the camera position or angle may cause a significant change in the input buffers with a strong impact on label positioning.

In this section we discuss approaches to smoothly adjust label positions when the user explores the scene. We employ two strategies to achieve the temporally coherent movement of the labels.

#### 3.5.1 Biasing Towards Previous Results

A popular strategy to ensure that the label position will not be significantly different between successive frames is to steer the algorithm of label placement towards choosing the same (or a similar) label position as in the previous frame. However, the algorithm should not prefer positions of the labels from the last frame if this would result in a labeling that is unfavorable with regards to the other label placement criteria. In our method, the preference for the previous frame’s label positions is expressed by the temporal coherence criterion *C*_4_. As the aggregation of the individual criteria in Eq. (6) is non-compensating, the temporal coherence criterion cannot compensate for deficiencies concerning the other criteria. Therefore, label positions close to the label positions in the last frame will not be selected if one of the criteria *C*_1_, *C*_2_, or *C*_3_ is bad for such positions in the current frame.

This strategy results in labels with a floating behavior, i.e., the labels seem as if they are attached by springs to the tagged objects, with occasional abrupt changes of their positions. The floating appearance of the labels might distract the user during heavy interaction with the scene. To further stabilize the movement of the labels, we use a second strategy during interaction, i.e., *label anchoring*.

#### 3.5.2 Label Anchoring in 3D

The approaches described in Section 3.1 and Section 3.2 produce 2D label positions as output. If we would use this output in each frame, upon user interaction the labels would slightly move around in a jittering motion. This would happen even if we incorporate the temporal coherency strategy described in Section 3.5.1.

The idea behind label anchoring to further stabilize the movement of the labels is straightforward. When the camera changes, instead of calculating the labels of the current frame, the computed labels from the last frame before the interaction are “anchored” at their 3D positions on the surface of the model. As described in Section 3.3, we determine the 3D position of the label on the surface of the annotated model. During interaction, we re-use the anchored 3D positions and perform the label rendering as described in Section 3.4 with the anchored labels instead of newly computing them. A similar approach for external labels has been introduced by Tatzgern et al. [47].

#### 3.5.3 Label Transitions

In this section we describe label transitions. These occur if a new label appears in the current frame, if an old label disappears in the current frame, and even if a label exists in both the previous and the current frame but on different positions. Such a situation occurs when the user stops interacting with the scene. Then, we need to make transitions from the *set of anchored labels* to the *set of the new labels* calculated for the current frame.

**Fig. 7:**
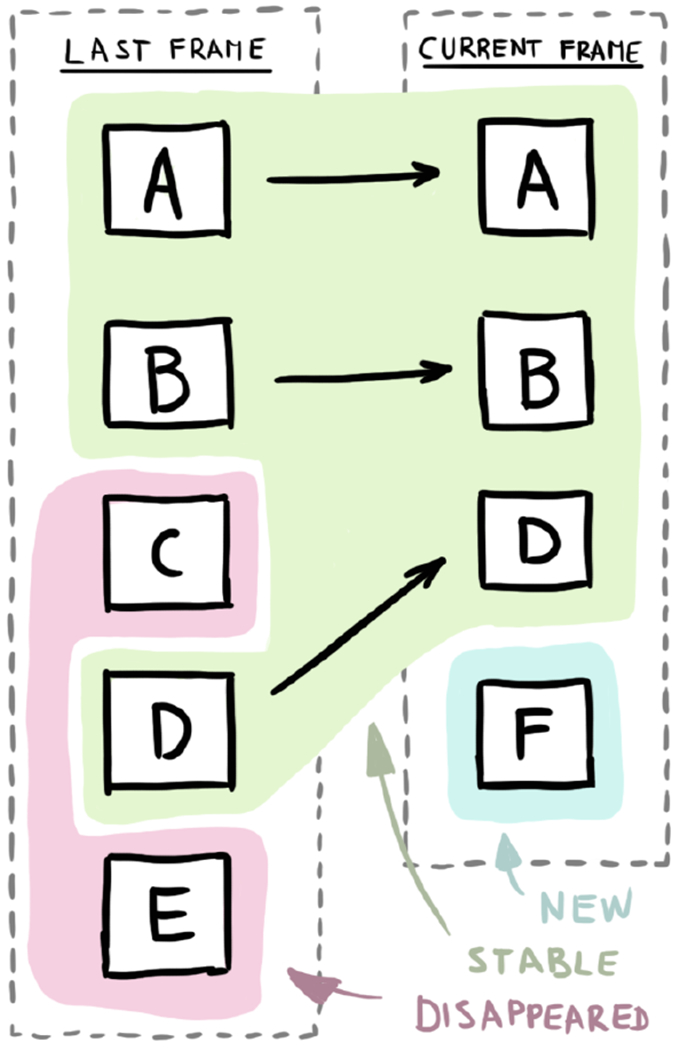
Matching of labels across two frames.

We examine the differences between the two label sets and categorize the labels in the two sets into three categories (see Figure 7):

**New label** is in the set of new labels and is not present in the set of anchored labels.

**Stable label** is in the set of new labels and also in the set of anchored labels.

**Disappeared label** is in the set of anchored labels and is not in the set of new labels.

By making this distinctions, we can communicate the changes in the state of the labels. New labels are not shown right away, but instead, they are faded-in using the alpha component of the label colors. Similarly, the disappeared labels are not hidden immediately, but they are faded-out slowly. The transitions for stable labels include an animation where the labels travel to their new positions over the course of few consecutive frames. These labels however should not be far away from their anchored positions due to the biasing towards previous results. This movement can be customized using the easing function to provide a more coherent perception.

**Fig. 8:**
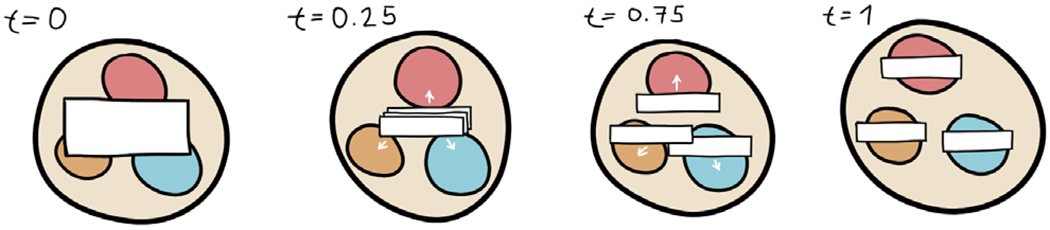
Transition from one parent label on a high level to several child labels on a lower level.

There are further possibilities for transitions between the two label sets. For example, if a parent label crosses a scale level, several child labels should be rendered instead of the parent label. An animated transition that highlights this relationship can be performed, as illustrated in Figure 8.

## 4 Results and Limitations

An implementation of the presented technique has been realized in the Marion framework for communicating biology [31]. We specifically extended the part of Marion dealing with large models from mesoscale biology. Real-time performance has been achieved using a modern graphics card (NVIDIA Titan X).

We demonstrate the benefits of labels on levels with a large scene representing an HIV virion immersed in blood plasma. The scene consists of more than 30k protein instances from 39 distinct types (excluding the membrane lipids). The virion consists of the envelope, made up by a lipid bilayer, and an inner matrix, formed by proteins and a capsid. The capsid envelope consists of pentamer and hexamer complexes, formed by proteins, and the capsid inner part contains proteins and the RNA fibre. Especially labeling an instance of both a hexamer and a pentamer is important, as these complexes look alike and might be considered the same on a first sight.

**Fig. 9:**
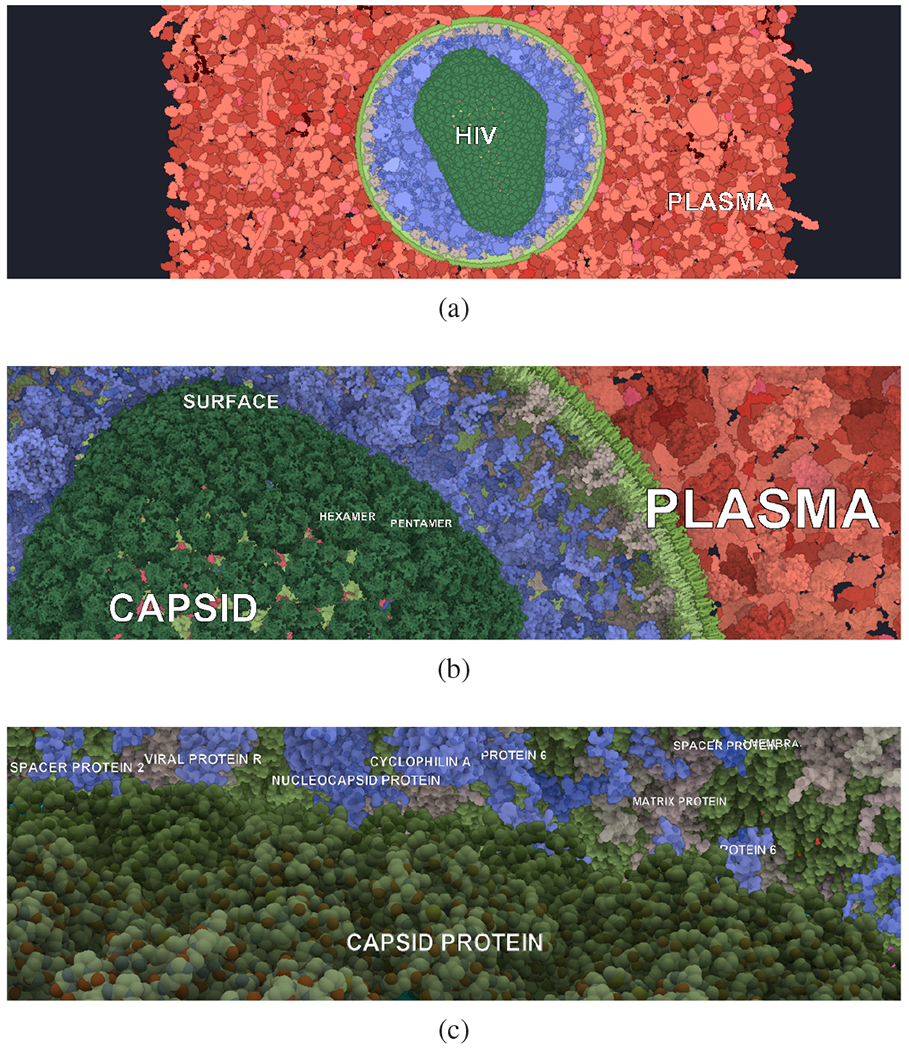
Labeling the capsid of the HIV virion. The capsid is one of the inner compartments of the virion, protecting the RNA fibre. The capsid surface is composed of hexamers and pentamers, which in turn consist of capsid proteins.

The multi-scale hierarchy of this scene contains six scale levels, where we exclude the root from labeling. The root of the hierarchy consists of two parts, i.e., the blood plasma and the HIV virion. Each of these contains a different number of hierarchical layers. Whereas the blood plasma consists only of one additional scale level containing all its proteins, the HIV virion consists of five different scale levels. Figure 9 illustrates a part of this hierarchy with the corresponding labeling. It also clearly shows that the hierarchy level corresponds to the label size. The interaction with the scene and transitions between labels are demonstrated in the supplementary video.

Strict thresholding for categorizing pixels into levels based on depth produces labels that pop in and out frequently if objects cross the threshold during interaction. This could be solved by processing the multi-scale regions by a mathematical morphology operator. With morphological closing, certain small regions that leak into the depths on another level will be removed and the threshold value is that way used in a more fuzzy way. In order to improve the temporal coherence hysteresis threshold could be applied as well.

Further, the labels occasionally overlap and thus readability is reduced. This is due to the approximation of labels as point data in our algorithm. Such an approximation is insufficient especially for labels containing longer text. To eliminate the overlaps, we need to handle the labels as rectangles instead of points. Another limitation of the current solution is that a label occludes the tagged object itself. This can be improved by enhancing the interaction. If the user hovers over the label, it is automatically repositioned to reveal the underlying tagged object of interest.

## 5 Discussion

We showed labels on levels (LoL) applied on mesoscopic biological data to our collaboration partners from structural biology. In general, their feedback has been positive, indicating that in structural molecular biology there are few labeling methods available. Little formal work has been done to improve on those. Nowadays, there are many efforts to generate multi-scale data and to assemble decades worth of results into whole cell models. Our collaborating biologists believe that labeling will be important in annotating these models.

A lot of attention in our discussions with domain experts has been dedicated to the communication of scale through label size. It seems to be a hit-or-miss situation, whether people understand this relationship or if they are confused by the double mapping (hierarchy level, perspective foreshortening) described in Section 3.4. This is an issue we will definitely need to address in future work.

There are now projects that build models ranging from whole organs, such as the human brain (with decimeters in size), down nine orders of magnitude to the atoms in the molecules making up the brain cells. We need to adapt the current visualization and labeling techniques to these massive multi-scale characteristics. Already a mesoscale model is posing visual problems that are difficult due to the model’s hierarchical structure, size, and complexity. The rather simple labeling methods available in most molecular viewers are of little use. Labels on levels is the first attempt to simultaneously annotate objects at various scale levels. This presents the opportunity that the method will be an essential tool in scale-dependent exploration, providing identification cues that work in concert with more general perceptual hints, such as color and molecular shape.

## 6 Conclusion and Future Work

In this paper, we present labels on levels, a novel approach to annotate large and dense hierarchical environments with multi-scale and multi-instance characteristics. Our labeling algorithm considers the level on which objects should be tagged and is, to the best of our knowledge, the first of its kind. We describe the entire sequence of steps that has to be processed in annotating such datasets. Labels on levels should be easily reproducible in other application areas as well. It works as a post-processing screen-space effect using only the structures that are typically available from the rendering of the scene itself.

In the future we intend to use the labels as an interface between the user and the data, acting as interactive elements to navigate in 3D space and across label levels. This will extend labels from being passive unidirectional communication tools to bidirectional interaction and communication widgets. Augmenting labels with interaction components will provide functionality to navigate across scales, hierarchies, and instances. For storytelling the camera path could be automatically determined as well as the visibility of elements that might either be related to an object of interest or on the contrary might occlude the focus structure and should be removed from the visualization. In this way the labels can also be used to regulate cutaway view settings. Including support for dynamic scenes will increase the labeling complexity and can be an interesting continuation of our work. Lastly, an interesting extension would be to associate audio data with the labels, so that the user can experience a guided audio-tour through a complex and annotated scene.

## Supplementary Material

Suppl Video

## Figures and Tables

**Fig. 1:**
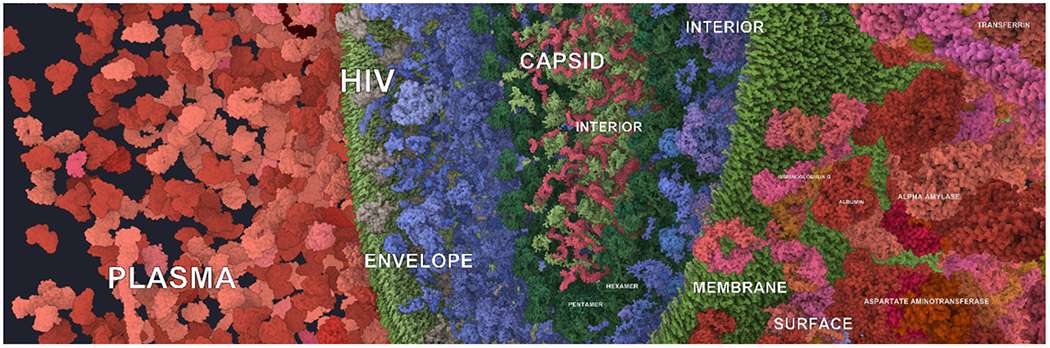
Multi-scale and multi-instance labeling of an HIV dataset that has thousands of copies (instances) of approximately 60 unique geometries: the foreground objects are automatically selected representative instances annotated with protein types, while the background objects are labeled with names of whole compartments, representing higher levels of the hierarchy.
